# Pheochromocytoma/paraganglioma-associated cardiomyopathy

**DOI:** 10.3389/fendo.2023.1204851

**Published:** 2023-07-13

**Authors:** Alicja Szatko, Piotr Glinicki, Małgorzata Gietka-Czernel

**Affiliations:** ^1^ Department of Endocrinology, Centre of Postgraduate Medical Education, Warsaw, Poland; ^2^ EndoLab Laboratory, Centre of Postgraduate Medical Education, Warsaw, Poland

**Keywords:** pheochromocytoma, paraganglioma, dilated cardiomyopathy, hypertrophic cardiomyopathy, takotsubo cardiomyopathy

## Abstract

Pheochromocytoma/paraganglioma (PPGL) are neuroendocrine tumors that frequently produce and release catecholamines. Catecholamine excess can manifest in several cardiovascular syndromes, including cardiomyopathy. PPGL-induced cardiomyopathies occur in up to 11% of cases and are most often associated with an adrenal pheochromocytoma (90%) and rarely with a paraganglioma derived from the sympathetic ganglia (10%). PPGL-associated cardiomyopathies can be chronic or acute, with takotsubo cardiomyopathy being the most often reported. These two types of PPGL-induced cardiomyopathy seem to have different pathophysiological backgrounds. Acute catecholaminergic stress inundates myocardial β-adrenoceptors and leads to left ventricle stunning and slight histological apoptosis. In chronic cardiomyopathy, prolonged catecholamine exposure leads to extended myocardial fibrosis, inflammation, and necrosis, and ultimately it causes dilated cardiomyopathy with a low ejection fraction. Sometimes, especially in cases associated with hypertension, hypertrophic cardiomyopathy can develop. The prognosis appears to be worse in chronic cases with a higher hospital mortality rate, higher cardiogenic shock rate at initial presentation, and lower left ventricular recovery rate after surgery. Therefore, establishing the correct diagnosis at an early stage of a PPGL is essential. This mini-review summarizes current data on pathophysiological pathways of cardiac damage caused by catecholamines, the clinical presentation of PPGL-induced cardiomyopathies, and discusses treatment options.

## 1 Introduction

In recent years, we have witnessed remarkable progress in understanding the rare type of neuroendocrine tumors that produce catecholamines, which arise from chromaffin cells within the adrenal medulla (pheochromocytoma) and extra-adrenal sympathetic ganglia (paraganglioma), collectively referred to as pheochromocytoma-paraganglioma (PPGL). According to the 5th series of the World Health Organization (WHO) Classification of Endocrine and Neuroendocrine Tumors, the term paraganglioma is used for both neoplasms, while pheochromocytoma is classified as an “intra-adrenal paraganglioma” (1). The annual incidence of PPGL increased during the past two decades, reaching an annual incidence of approximately 8 cases per million, mainly due to the increase in the number of imaging studies conducted in clinical practice (2). Despite higher awareness among clinicians and widely used imaging procedures, there are still PPGL cases that remain undetected, often leading to the fatal consequences of catecholamines excess, mainly cardiovascular complications (3).

The effect of supraphysiological levels of epinephrine and norepinephrine on the heart and vessels highly depend on the secretory profile of the PPGL (adrenergic vs. noradrenergic phenotype, episodic via. continuous release) (4). The most common, yet alone nonspecific sign of PPGL: hypertension, among other factors, may lead to myocardial hypoxia with various clinical manifestations: acute (takotsubo, ischemic) and chronic (dilated, hypertrophic) cardiomyopathy (5–8). Catecholamine-induced cardiomyopathy in PPGL (CICMPP) is potentially fatal, but an uncommon complication, with a prevalence of 8–11% of patients with a PPGL (9, 10). Although CICMPP is rarely the initial manifestation of PPGL, it is obligatory to rule out PPGL in patients with heart failure and paroxysmal symptoms: profuse diaphoresis, headaches, pallor, tremor, palpitations, and episodic hypertension, but also hyperglycemia in young patients with normal body mass index (BMI) (4, 10–12).

This review summarizes the pathophysiology of CICMPP, risk factors, the clinical presentation of takotsubo, dilated and hypertrophic CICMPP, treatment options, and future perspectives regarding recent advances in PPGL.

## 2 Pathophysiology of catecholamine-induced cardiomyopathy in PPGL

To understand the mechanisms leading to the diverse clinical manifestations of CICMPP, it is essential to comprehend how catecholamines affect the cardiovascular system. Catecholamines (epinephrine, norepinephrine, and dopamine) are tyrosine-derived hormones, and neurotransmitters synthesized predominantly in the adrenal medulla, sympathetic nerves, and brain (13, 14). Norepinephrine and epinephrine bind to adrenoceptors, while the effect of dopamine on adrenoceptors is negligible, but in high levels, dopamine may lead to hypotension mainly due to interaction with dopamine receptors present in mesenteric and renal vascular beds (15–17).

Adrenoceptors are a family of transmembrane G protein–linked receptors. There are two main types of adrenoceptors: α and β, which are further divided into nine subtypes: α1A, α1B, α1D, α2A, α2B, α2C, β1, β2, and β3. Binding the physiological ligands (norepinephrine and epinephrine) to adrenoceptors result in G protein–mediated transduction of the signal, activation of second messengers or ion channels, evoking a response in the cell, highly dependent on the type of adrenoceptor and target tissue (18).

α1-adrenoceptors are mostly found in vascular smooth muscle but are also present in the myocardium (subtype α1A) (19, 20). α1-adrenoceptors signal transduction leads to protein kinase C (PKC) activation, 1,4,5-inositol triphosphate production, and intracellular calcium flow (6, 19). This results in smooth muscle contraction, and increased cardiac output, while persistent α1-adrenoceptors stimulation leads to the hypertrophic phenotype (6, 19). α2-adrenoceptors can be found in vascular smooth muscle distally from the sympathetic nerve (mainly α-2B subtype), leading to vasoconstriction, while presynaptic α2-adrenoceptors inhibit norepinephrine release resulting in the reduction of the sympathetic stress response (20, 21). β1-adrenoceptors are expressed in the sinoatrial node, atrioventricular node, and cardiomyocytes, resulting in calcium-mediated increased contractility, heart rate, and enhanced conduction of electrical stimulus (20). In the cardiovascular system, stimulation of β2-adrenoceptors, through inhibition of cAMP production, leads to vasodilatation and relaxation of the myocardium (20, 22).

Norepinephrine has a stronger affinity to α-adrenoceptors than β-adrenoceptors, leading to increased cardiac output and vasoconstriction, which manifests as elevated blood pressure (6). Early response to norepinephrine includes a rise in heart rate, but activation of the baroreflex by the increased blood pressure causes the heart rate to decrease (6). Considering the affinity of norepinephrine to adrenoceptors and the typically continuous pattern of catecholamine release, persistent hypertension and arrhythmias are part of the clinical presentation (22–24). Epinephrine binds to all major adrenoceptors: α1, α2, β1, and β2 (20). At low concentrations, epinephrine is selective for β2-adrenoceptors. The ability to stimulate peripheral β2-adrenoceptors manifests in patients with a PPGL secreting epinephrine as hypotension, once α1-adrenoceptors are pharmacologically blocked (20, 22, 25, 26). At higher concentrations, epinephrine stimulates α-adrenoceptors resulting in vasoconstriction (20). Epinephrine at higher concentrations also has a strong affinity for β1-adrenoceptor, which results in positive inotropic, chronotropic, and dromotropic effects (20). Typically, a PPGL secreting epinephrine in an episodic release pattern is often experienced by the patients as tachyarrhythmias (22, 25, 26). Furthermore, life-threatening, excessive amounts of catecholamines lead to vasoconstriction (including coronary arteries), myocardial ischemia, and necrosis (27). Conditions (e.g., hyperthyroidism, hypercortisolism, hypokalemia, hypocalcemia) that increase the expression and sensitivity of the adrenoceptor amplify the devastating impact of catecholamine excess (3, 28–30).

Acute, uncontrolled release of catecholamines in PPGL causes hyperstimulation of β1-adrenoceptors, increasing heart rate and contractility (31). Furthermore, it significantly raises myocardial oxygen demand, especially when combined with coronary artery spasms due to the activation of α1-adrenoceptors, exposing the myocardium to hypoxia (31). Catecholamine surge also leads to microvascular alterations combined with calcium overload, which according to Wittstein et al., may contribute to reversible coronary vasoconstriction, as observed in patients with takotsubo CICMPP (6, 32).

Myocardial cytosolic and mitochondrial calcium overload, one of the most prominent features of persistent catecholamine excess, promotes oxidative stress and mitochondrial permeability, leading to cell death (33). Mitochondrial calcium excess promotes hydrogen peroxide synthesis during oxidative deamination of catecholamines (31). The production of superoxide anion radicals is also enhanced by α1-adrenoceptor stimulation: they are products of nicotinamide adenine dinucleotide phosphate (NADPH) reactions (34). Another process that contributes to catecholamine-induced oxidative stress in cardiomyocytes is auto-oxidation of catecholamines producing highly reactive, toxic, and unstable “aminochromes” and inactive, more stable “aminolutins” which can be measured in the plasma (6, 35, 36). The reaction is accelerated by oxygen free radicals and various enzymes, e.g., myeloperoxidase, cytochrome oxidase (6). The toxicity of oxidized catecholamines was proven in the study of Yates et al., in which perfusion with oxidized isoproterenol in isolated rat hearts was found to induce ultrastructural mitochondrial damage in cardiomyocytes — the phenomenon was not observed when fresh isoproterenol was used (37). Furthermore, adrenochrome (50 mg/L) infusion of isolated rat hearts ceased contractile activity in 30 minutes, whereas epinephrine or metanephrine infusions had a positive impact on cardiac contractile activity (38). However, studies concerning the role of “aminochromes” in CICMPP are still missing.

Chronic exposure to catecholamine excess leads to desensitization of β-adrenoceptors (39). Desensitization occurs through the phosphorylation of the adrenoceptor by the G protein–coupled receptor kinase (GRK) and binding to the protein called β-arrestin2. GRK2/β-arrestin2 complexes promote β1-adrenoceptor uncoupling and internalization (40). Furthermore, high catecholamine stress causes β2-adrenoceptors to switch from the Gs to Gi signaling pathway, leading to decreased cardiac contractility (41). Interestingly, not only is the density of β2/β1-adrenoceptors higher at the apex than at the base, but also apical adrenoceptors show higher sensitivity to catecholamines than those at the base (41). The apex–base gradient of β-adrenoceptors and described switch from Gs to Gi of β2-adrenoceptors (also mainly observed in the apex) explain the impaired regional contractility and typical clinical presentation of takotsubo cardiomyopathy: apical hypokinesia and ballooning (41). The negative inotropic effect depends on β2-adrenoceptor phosphorylation by both protein kinase A (PKA) and GRKs (42). It is noteworthy that L41Q GRK5 polymorphism is associated with enhanced desensitization and impaired β-adrenoceptor response, and it is more prevalent among patients with takotsubo cardiomyopathy (43).

## 3 Predisposing factors and clinical presentation

Given the fact that one in ten patients with PPGL will develop CICMPP, a potentially fatal complication, it is essential to identify and closely monitor predisposed individuals. The results of the study by Wang et al. on 50 patients with CICMPP and 152 patients with PPGL without diagnosed CICMPP, identified five risk factors for CICMPP, namely maximum resting heart rate ≥ 115 per minute, maximum resting systolic blood pressure ≥ 180 mmHg, blood glucose ≥ 8.0 mmol/L, 3 or more reported symptoms (headache, sweating, hypertension, hypotension, palpitation, chest pain, dyspnea, impaired tip perfusion, syncope, nausea, and vomiting), and early onset ≤ 40 years (44). Interestingly, in the study by Zhao et al., among patients with PPGL, female sex, paroxysmal symptoms, PPGL secreting more than one catecholamine, and higher white blood cell and platelet counts were significantly more prevalent in patients developing cardiovascular complications (45). The link between the higher cardiovascular risk in patients with PPGL and increased platelet count may be explained by catecholamine-mediated modulation of platelet function and aggregation via stimulation of dopaminergic and α2-adrenoceptors expressed on platelets, since the activation of platelets is crucial in the pathogenesis of various cardiovascular diseases, e.g., hypertension and atherosclerosis (46–51). Genetic biomarkers may also help to determine the risk of CICMPP. In the recent study by Amar et al., the α2-adrenoceptor variant (alpha 2CDel322–325) was more prevalent among patients with CICMPP (52).

### 3.1 Takotsubo cardiomyopathy

Excess of catecholamines, apart from other stress factors, may lead to acute, reversible left ventricular wall motion abnormalities (LVWMA) with a regional or circumferential pattern extending beyond the coronary artery supply, named after the Japanese fishing pot used to catch octopus — takotsubo cardiomyopathy (53–56). LVWMA in takotsubo cardiomyopathy results in characteristic left ventricle ballooning during systole (56). LVWMA may affect the apical, mid-apical, mid-ventricular, mid-basal, and basal segments of the left ventricle (57). The clinical presentation of takotsubo CICMPP does not differ from acute coronary syndrome: patients usually report chest and/or abdominal pain, dyspnea, and the majority of patients also experience symptoms that should raise the suspicion of PPGL (e.g., palpitations, profuse sweating, headache) (53). The most common electrocardiogram (ECG) changes in takotsubo CICMPP include ST-elevation myocardial infarction (STEMI)–like changes (more than one-third of the patients), ST segment depression, T-wave inversion, and QT-prolongation (53, 58). Although elevated troponin concentrations were observed in 71–95% of patients with takotsubo cardiomyopathy, the peak values in myocardial infarction biomarkers are usually lower compared to patients with acute coronary syndrome and not proportional to left ventricle impairment (53, 58, 59).

On echocardiography, apical ballooning, hypokinesia, akinesia, dyskinesia of apical segments, and the occasional hypokinetic mid-segments, are the classic presentation of takotsubo cardiomyopathy (6, 59). However, it occurs also in reversed (inverted) form when basal segments are akinetic, while the apex is hyperkinetic (6, 59–61). The latter phenotype is rare, yet more prevalent in patients with takotsubo CICMPP than in the overall group of takotsubo cardiomyopathy patients: 28.8% vs. 2.2% in the meta-analysis presented by Y-Hassan et al. (62). Moreover, a global pattern is more frequently present in takotsubo CICMPP (62). Compared to the overall takotsubo cardiomyopathy group, takotsubo CICMPP was characterized by higher complication rates: 68.2% vs. 21.8% (i.e., heart failure, pulmonary edema, and cardiogenic shock) and recurrence rate, whereas mortality was reported in 4% of cases and did not differ between the groups (62). In the takotsubo CICMPP group, death occurred in 7% of men and 2.7% of women (p = 0.35) (62). Mortality increased significantly during the recurrence of takotsubo CICMPP (11%, 2/18) (62).

### 3.2 Hypertrophic cardiomyopathy

Longstanding hypertension in undetected PPGL may lead to left ventricle outflow tract obstruction: a hallmark of hypertrophic CICMPP. Patients with hypertrophic cardiomyopathy usually report exertional dyspnea and fatigue with or without chest pain or presyncope (6, 63). In advanced stages, patients may experience orthopnea and/or fluid retention with peripheral/pulmonary edema (63). The typical symptoms of patients with hypertrophic CICMPP may also be complemented with PPGL-suggestive symptoms, namely profuse sweating, and palpitations (64, 65). ECG alterations often meet the criteria of left ventricle hypertrophy (64). Echocardiography shows systolic anterior motion of the anterior mitral valve leaflet, increased left ventricle outflow tract gradient with persisted ejection fraction (EF), and septal and posterior wall hypertrophy (64, 66, 67).

Interestingly, the results of the study by Dobrowolski et al. prove that subclinical impairment of systolic function in PPGL patients was independent of the presence of left ventricle hypertrophy (LVH) (68). In the abovementioned study, the assessment included a global longitudinal strain (GLS) — a parameter derived from two-dimensional speckle-tracking echocardiography — allowing to assess the function of longitudinally-oriented subendocardial fibers, which are the most susceptible to ischemia and wall stress. GLS more accurately reflects intrinsic myocardial function and early systolic dysfunction than EF (68, 69). The patients with PPGL had lower GLS (median 17.2%) than in the control group, while EF did not differ significantly (68). Early systolic dysfunction was confirmed in the meta-analysis, including 252 patients with PPGL, speckle tracking echocardiography (STE) revealed worse GLS in the pooled PPGL group when compared to the control group (−17.3 ± 1.2 vs. −20.0 ± 0.6), differences in EF were not observed between the groups (70). In the study by Dobrowolski et al., the adrenergic biochemical phenotype was associated with worse systolic function and nonsignificantly higher left ventricle mass index compared to BP-matched controls, indicating that apart from pressure overload, epinephrine per se may contribute to LVH (68). Experimental findings suggested the role of catecholamines in the induction of protein synthesis (71). Both systolic and diastolic alterations in patients with PPGL reversed significantly after curative surgery (68).

### 3.3 Dilated cardiomyopathy

Patients with dilated cardiomyopathy typically experience symptoms of progressive systolic dysfunction, often after a latent period when they are clinically asymptomatic (72, 73). Interestingly, in the analysis by Zhang et al., among all CICMPP cases, PPGL associated with genetic syndromes or metastatic PPGL were found predominantly in patients with dilated PPGL (23% of cases, i.e., multiple endocrine neoplasia type 2, neurofibromatosis type 1, and von Hippel-Lindau syndrome) (72). However, the abovementioned retrospective study has several limitations, including incomplete data regarding imaging findings (e.g., missing echocardiographic parameters leading to the categorization of 14 cases under unspecified cardiomyopathy) and the results of molecular analysis (e.g., SDHB mutations were not included) (72). In dilated CICMPP, ECG may be normal, although alterations ranging from T wave changes and left bundle branch block to disturbances in atrioventricular conduction may occur (73). Echocardiography reveals increased left ventricle end-diastolic volumes or diameters (> 2 Standard Deviations (SDs) from normal) with global systolic dysfunction not attributable to ischemic or valvular disease (73). Cardiovascular magnetic resonance (CMR) shows left ventricle dilatation, and it is also of use to rule out inflammatory processes, to assess rest stress myocardial perfusion, myocardial perfusion, iron, and fat deposition, and aortic distensibility in CICMPP (73, 74). The key to the successful management of dilated CICMPP is complete PPGL resection, which leads to the improvement of EF and lower mortality rate: death occurred in 4% (2/52) of patients who underwent surgical resection of a PPGL and 22% (2/9) of patients not treated surgically (72). The importance of precise screening and prompt diagnosis is highlighted by two case reports of heart transplants undertaken before the diagnosis of PPGL was established (75, 76).

**Figure 1:**
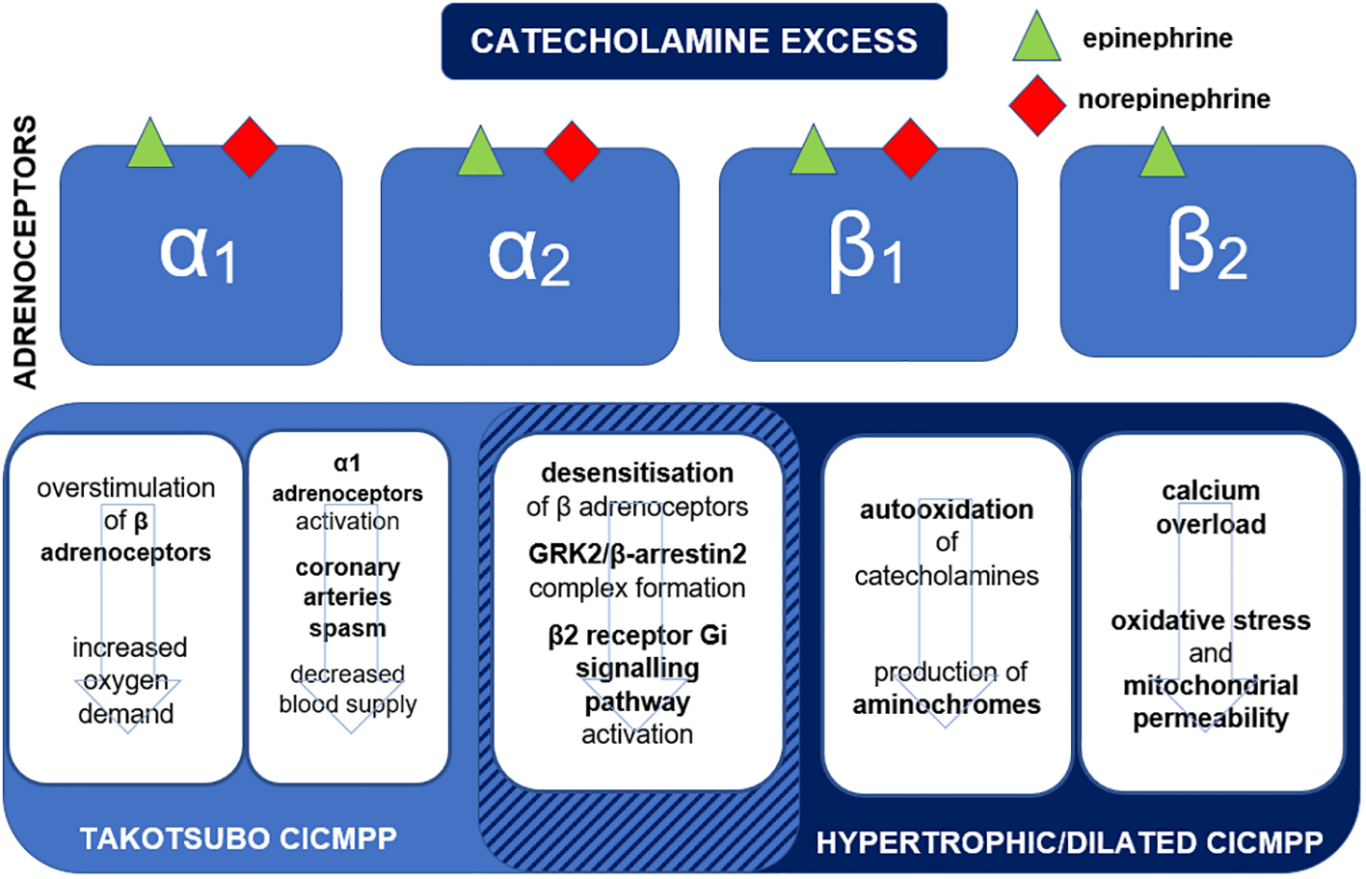
Binding affinities of epinephrine and norepinephrine to adrenoceptors and mechanisms leading to the development of subtypes of catecholamine-induced cardiomyopathy in pheochromocytoma-paraganglioma (CICMPP). G_i_, inhibiting G protein; GRK, G protein–coupled receptor kinase.

The catecholamine binding affinities to adrenoceptors and pathophysiologic mechanisms leading to different subtypes of CICMPP are presented in **Figure 1**.

## 4 Treatment

The early diagnosis, confirmed by biochemical tests (elevated free or fractionated plasma or urine metanephrines), followed by localization of the lesion and complete PPGL resection is the mainstay of the successful treatment of patients with CICMPP. The analysis by Zhang et al. showed that PPGL resection was associated with an improved EF in 96% of CICMPP cases (72). Prior to surgery, α-adrenoceptor blockade should be initiated for 7–14 days (77). Some studies favor α1-selective over nonselective α-adrenoceptor blockers due to lower preoperative diastolic pressure, lower intraoperative heart rate, and better postoperative outcome (77, 78). However, the results of the randomized controlled PRECIST trial showed no differences between phenoxybenzamine and doxazosin in the duration of blood pressure being outside the target range during operation, but phenoxybenzamine was more efficient in preventing intraoperative hemodynamic instability (79). The addition of metyrosine (tyrosine hydroxylase inhibitor) should be considered for patients at high risk of catecholamine surge (e.g., with symptomatic, multifocal, or metastatic disease intolerance of α-adrenoceptor blockers or when difficult surgery of PPGL encroaching neighboring vascular structures is anticipated) (80). However, the availability of metyrosine is limited (11).

Once the α-adrenoceptor blockade is assured and the target heart rate (of 60–70 bpm seated and 70–80 bpm standing) is not achieved, a β-adrenoceptor blocker should be initiated (not earlier than 3–4 days after initiation of an α-adrenoceptor blocker) (22, 77). Selective β1-adrenoceptor blockers are favored (since β2-adrenoceptor blockade may result in hypertension). In the emergency setting, intravenous fast-acting esmolol or alternatively metoprolol may allow to optimally react to hemodynamic alterations (3). Among oral β-adrenoceptor blockers, metoprolol succinate (controlled-release) or atenolol are preferred (3). The choice of β-adrenoceptor blockers in the treatment of CICMPP should also include subtype-specific recommendations: preferred non-vasodilating β-adrenoceptor blockers (atenolol, metoprolol, bisoprolol) in hypertrophic cardiomyopathy (81).

The preoperative aim is a blood pressure of less than 130/80 mm Hg while seated and greater than 90 mm Hg systolic while standing (77). If blood pressure is not optimally controlled calcium channel blockers may be added (77). If heart failure is confirmed, angiotensin-converting enzyme inhibitors (ACEI) or angiotensin receptor blockers (ARBs) are a part of pharmacological therapy. There are also novel, potential therapeutic options for patients with PPGL cardiomyopathy and heart failure, namely sodium-glucose cotransporter 2 inhibitors (SGLT2i) and angiotensin receptor/neprilysin inhibitor (ARNI). Multiple trials proved their efficacy in patients with heart failure, but in PPGL data are limited and further studies are needed (82, 83). Before the resection of PPGL, a high-sodium diet and fluid intake should be assured to prevent severe hypotension after PPGL removal (77). Preoperative assessment should also include an electrocardiogram and echocardiogram, which may identify the features of CICMPP (84).

Patients with PPGL are more prone to develop acute cardiovascular complications. In case of hypertensive crisis, intravenous administration of phentolamine, sodium nitroprusside, or nicardipine should be initiated. Once the state of the patient is stable, titration of the phenoxybenzamine dosage can be initiated to reach the target blood pressure (85). Fluid status should be monitored, diuretics are to be avoided unless the patient has fluid congestion, and even then, administered judiciously (6). In the management of hemodynamic instability, vasoactive amines are often administered, but their efficacy may be limited due to sympathetic receptor down-regulation, and they may even exacerbate PPGL-induced cardiac dysfunction (6, 86–88). If hypotension persists despite pharmacological treatment, mechanical circulatory support (MCS) may be needed. In the systematic review of 62 patients with severe systolic dysfunction (median left ventricular ejection fraction (LVEF) of 16% (range 5–32%)) requiring extracorporeal life support (ECLS) due to intractable pheochromocytoma crisis, full recovery of left ventricle function (LVEF >50%) was observed in most patients and 54 (87%) of 62 reported cases survived (89). Also, there are reports of successful left ventricular assist device (LVAD) use in PPGL-induced heart failure and perioperative management (90–92). An intra-aortic balloon pump (IABP) has been used for unresponsive patients but was not effective (93).

**Figure 2:**
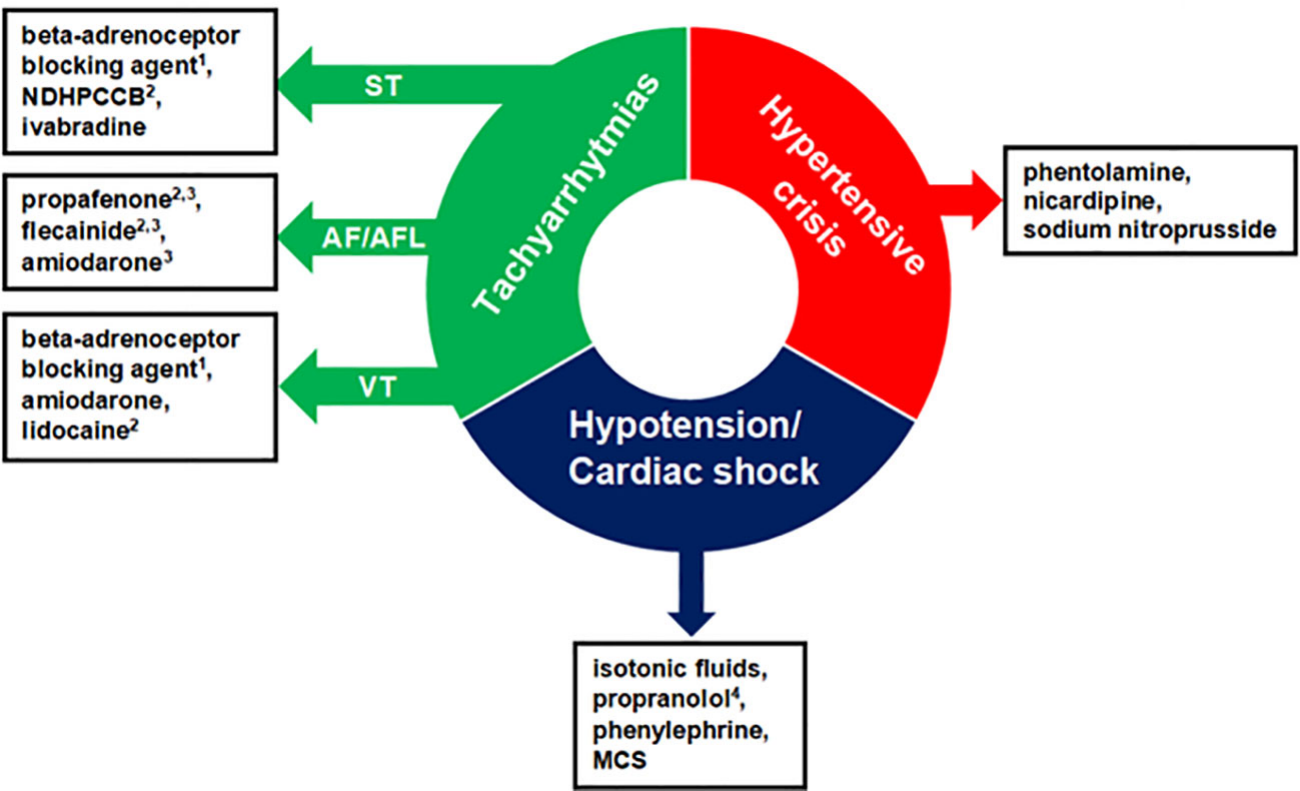
Pharmacological management of acute cardiovascular complications in patients with pheochromocytoma-paraganglioma. The order of presented drugs does not correspond to first-, second-, and third-line therapy and the choice of administered drugs should be individualized. ^1^With alpha-adrenoceptor blockade, otherwise, it can precipitate a hypertensive crisis. ^2^Antiarrhythmic agent not recommended in systolic dysfunction. ^3^Used to restore sinus rhythm. ^4^In case of hypotension and suspected beta2-adrenoceptor overstimulation. NDHPCCB, Nondihydropyridine Calcium Channel Blockers; ST, Sinus Tachycardia; AF/AFL, Atrial Fibrillation/Atrial Flutter; VT, Ventricular Tachycardia; MCS, Mechanical Circulatory Support.

The general pharmacological management of hypertensive crisis, hypotension/cardiac shock, and the most common tachyarrhythmias in PPGL are summarized in **Figure 2**.

## 5 Conclusions

CICMPP is a potentially fatal complication of PPGL, but there are still patients with CICMPP not diagnosed early enough. Thus, it is essential to broaden awareness about the clinical course and adequate management of CICMPP among clinicians and underline the importance of accurate cardiac assessment of PPGL patients.

Currently, the management of CICMPP is based on the guidelines for PPGL treatment, recommended general cardiological interventions, published case series, and few systematic reviews or meta-analyses. Dedicated guidelines of CICMPP management addressing specific features of this rare entity and integrating novel advances in pharmacotherapy and MCS could help to optimally treat patients with CICMPP.

Probably the ongoing progress in genetics and metabolomics in PPGL, completed by integration of the results using artificial intelligence, may contribute to a better understanding of the diverse effects of catecholamine excess on the cardiovascular system, identify predisposing factors (also among asymptomatic carriers of pathogenic mutations in genes predisposing to PPGL development), biomarkers, and establish the prognosis.

## Author contributions

MG-C provided the idea for the manuscript. AS, PG, and MG-C reviewed the relevant literature and drafted the manuscript. AS drafted the figures. MG-C and PG reviewed critically the manuscript. All authors have made substantial contributions to the manuscript and have read and approved the final version of the manuscript.
